# Gold catalysis for organic synthesis II

**DOI:** 10.3762/bjoc.9.241

**Published:** 2013-10-09

**Authors:** F Dean Toste

**Affiliations:** 1 Department of Chemistry, University of California, Bekeley, CA, USA

**Keywords:** gold catalysis

Two years have now passed since the publication of the first Thematic Series on gold catalysis for organic synthesis in the Beilstein Journal of Organic Chemistry. In the intervening time, the pace of progress and discovery in the field has continued unabated. Many of these advancements are exemplified in the more than twenty contributions that have been collected in this second Thematic Series.

Many of the early examples of homogeneous gold-catalysis focused on the ability of cationic gold complexes to activate π-bonds towards attack of nucleophiles. This reactivity platform, examples of which can be found in this Thematic Series, continues to provide a fruitful basis for the discovery of important new transformations. This includes the development of a tandem or domino process that can be employed for the synthesis of complex polycyclic structures. These articles demonstrate the power of gold catalysis for the construction of complex structures, including the development of tandem processes, the expedient synthesis of heterocyclic structures, and applications to the synthesis of complex natural products.

The field has also witnessed growth through the discovery of other modes of reactivity. For example, gold-catalyzed cycloaddition reactions, examples of which are found in this Thematic Series, have featured prominently. Additionally, enantioselective catalysis with gold has seen notable advancements and is highlighted in several articles. The remarkable breakthroughs in oxidative transformations catalyzed by gold complexes, including those employing *N*-oxides as stoichiometric oxidants, have featured prominently in many recent innovations. Taken together, these articles present an outstanding overview of the state of the field from many of its top practitioners. They foreshadow the many additional reactions, reactivity modes, and catalysts based on gold that remain to be uncovered. I am grateful to the authors for their excellent contribution and making this second Thematic Series as successful as the first.

F. Dean Toste

Berkeley, October 2013

